# Transcriptomics Sequencing Provides Insights into Understanding the Mechanism of Grass Carp Reovirus Infection

**DOI:** 10.3390/ijms19020488

**Published:** 2018-02-06

**Authors:** Geng Chen, Libo He, Lifei Luo, Rong Huang, Lanjie Liao, Yongming Li, Zuoyan Zhu, Yaping Wang

**Affiliations:** 1 State Key Laboratory of Freshwater Ecology and Biotechnology, Institute of Hydrobiology, Chinese Academy of Sciences, Wuhan 430072, China; chengeng@ihb.ac.cn (G.C.); helibowudi@ihb.ac.cn (L.H.); luolifei145@163.com (L.L.); huangrong@ihb.ac.cn (R.H.); liaolj@ihb.ac.cn (L.L.); liyongming8080@sohu.com (Y.L.); zyzhu@ihb.ac.cn (Z.Z.); 2 University of Chinese Academy of Sciences, Beijing 101408, China

**Keywords:** grass carp, grass carp reovirus, transcriptomics sequencing, phagosome

## Abstract

Grass carp is an important aquaculture fish species in China that is affected by severe diseases, especially haemorrhagic disease caused by grass carp reovirus (GCRV). However, the mechanisms of GCRV invasion and infection remain to be elucidated. In the present study, *Ctenopharyngodon idellus* kidney (CIK) cells were infected with GCRV, harvested at 0, 8, 24, and 72 h post infection, respectively, and then subjected to transcriptomics sequencing. Each sample yielded more than 6 Gb of clean data and 40 million clean reads. To better understand GCRV infection, the process was divided into three phases: the early (0–8 h post infection), middle (8–24 h post infection), and late (24–72 h) stages of infection. A total of 76 (35 up-regulated, 41 down-regulated), 553 (463 up-regulated, 90 down-regulated), and 284 (150 up-regulated, 134 down-regulated) differently expressed genes (DEGs) were identified during the early, middle, and late stages of infection, respectively. Gene Ontology (GO) and Kyoto Encyclopedia of Genes and Genomes (KEGG) enrichment analysis revealed that DEGs were mainly involved in carbohydrate biosynthesis, transport, and endocytosis in the early stage, phagocytosis and lysosome pathways were mainly enriched in the middle stage, and programmed cell death, apoptosis, and inflammation were largely associated with the late stage. These results suggest GCRV infection is a gradual process involving adsorption on the cell surface, followed by endocytosis into cells, transport by lysosomes, and eventually resulted in cell necrosis and/or apoptosis. Our findings provide insight into the mechanisms of grass carp reovirus infection.

## 1. Introduction

The grass carp (*Ctenopharyngodon idellus*) has been reared in China for more than 60 years as an important aquaculture species. The production of grass carp reached 5.8 million tons in 2015, accounting for more than 13% of the world’s freshwater aquaculture production [1]. Grass carp haemorrhage disease, caused by grass carp reovirus (GCRV), is one of the most damaging diseases, resulting in huge economic losses to the aquaculture industry of grass carp [2]. GCRV was first isolated in China and belongs to the genus *Aquareovirus* of the family *Reoviridae* [3]. GCRV not only infects grass carp but also infects the rare minnow (*Gobiocypris rarus*), black carp (*Mylopharyngodon piceus*) and topmouth gudgeon (*Pseudorasbora parva*), causing haemorrhagic symptoms and death in these species. 

Grass carp haemorrhage disease outbreaks are frequent and result in huge economic losses in the aquaculture industry, hence the GCRV is of particular interest to geneticists aiming to develop strategies for breeding disease-resistant fish. Recent studies on GCRV have mainly focused on identification of genes involved in immune responses to GCRV infection [4,5], cloning and characterization of GCRV-encoded proteins [6,7], the development of vaccines against GCRV [8,9], and transcriptomic sequencing of discovered immune-related genes or uncovered the antiviral immunity mechanism of grass carp infected with GCRV [10,11,12,13,14]. However, the mechanisms of grass carp reovirus invasion and infection remain poorly explored.

In the present study, *C. idellus* kidney (CIK) cells were infected with GCRV, harvested at 0, 8, 24, and 72 h post infection, respectively, and subjected to transcriptomics sequencing. Gene Ontology (GO) and Kyoto Encyclopedia of Genes and Genomes (KEGG) enrichment analysis were performed on differentially expressed genes (DEGs) identified during different stages of infection. Our findings expand our understanding of the mechanisms underlying grass carp reovirus infection.

## 2. Results

### 2.1. GCRV Infection Induces Cytopathic Effects

**Figure 1:**
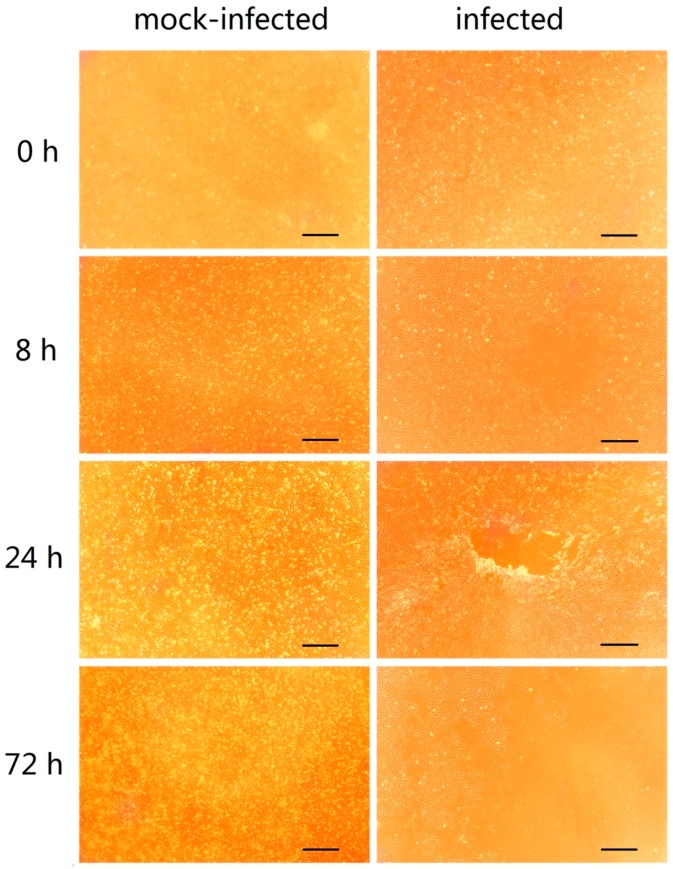
(*C. idellus* kidney) CIK cells mock-infected and infected with grass carp reovirus (GCRV). The images were adjusted for contrast to improve their appearance, scale bar represents 200 μm.

As shown in Figure 1, no cytopathic effect (CPE) was observed in mock-infected cells. However, in cells infected with GCRV, a CPE was observed as early as 8 h post infection, and it became more pronounced with over time. At 72 h post infection, it could be seen that most cells were necrotic.

### 2.2. Cell Counting Kit-8 Assay

**Figure 2:**
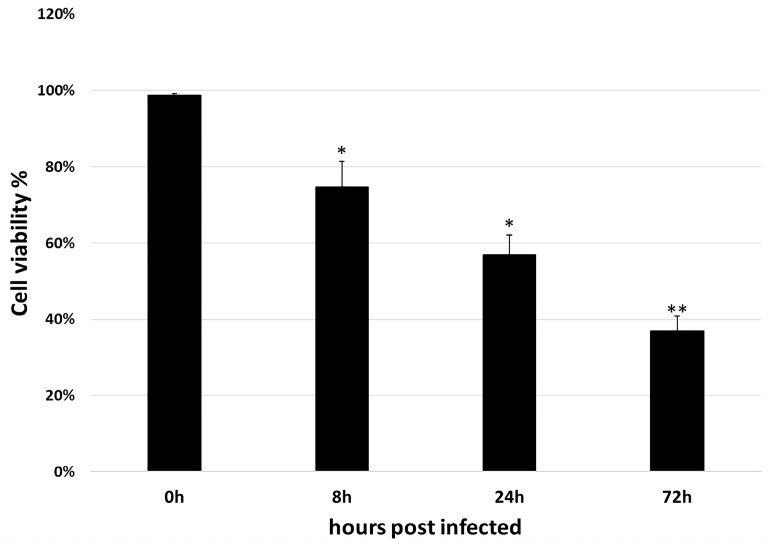
Cell viability assay after infected with GCRV. After 0, 8, 24 and 72 h post infection, cells were evaluated using CCK-8 assay, respectively. The mean of three replicates was shown with the ±standard deviation (S.D.). Significant difference between the control and treated group was indicated with asterisks (*: *p* < 0.05; **: *p* < 0.01)

As shown in Figure 2, the mock-infected cells showed the viability near 100%. However, for cells that infected for 8, 24, and 72 h, only 74.66%, 56.88%, and 36.95% of cell viability were observed. Therefore, the result suggested that GCRV infection is efficiency.

### 2.3. Preliminary Analysis of Transcriptomics Sequencing Data

**Figure 3:**
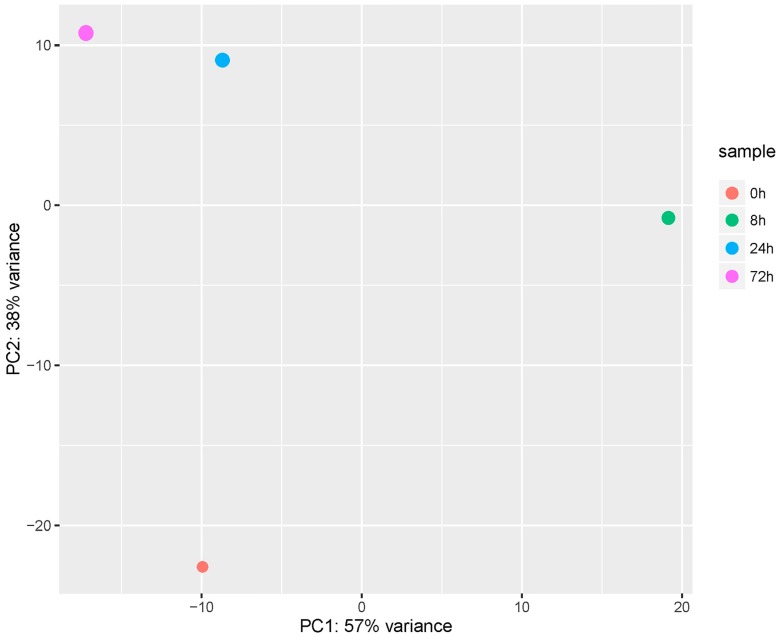
Principal Component Analysis. The PC1 indicates the differences among infected samples, whereas PC2 shows differences between control and infected samples.

As shown in Table 1, the raw reads, clean reads, clean bases, Q20, Q30, and mapped percentage were recorded for each library. For all libraries, Q20 ≥ 96%, Q30 ≥ 92% and mapped percentage ≥87%. These results showed the high quality of the sequencing data and ensured suitability for further analysis. All sequencing data have been uploaded to the Sequence Read Archive (SRA) of the National Center for Biotechnology Information (NCBI; accession number SRP111072). Moreover, principal component analysis (PCA) was performed on each sample according to the expression level. As shown in Figure 3, mock-infected cells were clearly distinct from cells infected with GCRV. For samples infected with GCRV, correlation values were proportional to the time post-infection; samples from 8 h did not cluster with those from 24 and 72 h. Therefore, the results suggested that infection efficiency and differences existed in each sample post infection.

### 2.4. Identification of Differently Expressed Genes (DEGs)

To better understand GCRV infection, the process was divided into three phases: early (0–8 h post infection), middle (8–24 h post infection), and late (24–72 h) stages. In each phase, data from the latter time point were compared with the former (8 vs. 0, 24 vs. 8, and 72 vs. 24) to identify differentially expressed genes (DEGs). As shown in Table 2, a total of 76 (35 up-regulated, 41 down-regulated), 553 (463 up-regulated, 90 down-regulated), and 284 (150 up-regulated, 134 down-regulated) DEGs were identified during early, middle, and late stages of infection, respectively. Detailed information related to these DEGs is shown in Table S1. DEGs that could not be functionally annotated are listed as ‘unknown’.

### 2.5. GO and KEGG Enrichment Analysis

Go enrichment analysis was performed to investigate the putative roles of DEGs. The early stage of infection was mainly related to iron transport, carbohydrate metabolism, and inclusion bodies. For the middle stage of infection, iron ion binding, lysosome categories were significantly enriched. Meanwhile, the late stage was mainly associated with metabolic processes such as lipid biosynthetic, steroid metabolic, and alcohol biosynthetic metabolic. The top five enriched GO terms in each of the three stages are listed in Table 3, and details of the GO terms are included in Table S2.

KEGG enrichment analysis was also performed for DEGs identified in the three stages. During the early stage of infection, DEGs were mainly involved in pathways related to focal adhesion, ECM (extracellular matrix)–receptor interactions, cytokine–cytokine receptor interactions. In the middle stage, pathways related to virus entry into cells and cell recognition were enriched, including the phagosome, lysosome, and hippo signaling pathways. In the late stage of infection, the most enriched pathways were the biosynthesis of steroids and the terpenoid backbone, and degradation of ketone bodies. The top five enriched KEGG terms in each of the three stages are listed in Table 4, and details of the KEGG terms are included in Table S3.

### 2.6. Expression of DEGs in Key KEGG Pathways

**Figure 4:**
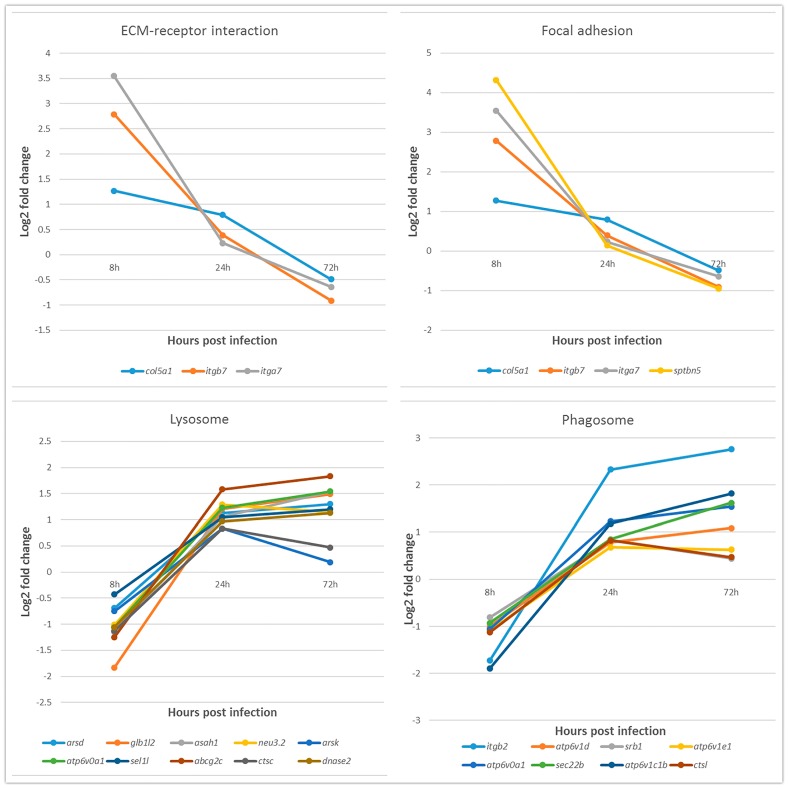
Line chart of gene expression patterns of differentially expressed genes (DEGs) in selected KEGG pathways. The x-axis is the hours post infection, and the y-axis is the log2 fold change in expression level. Lines of different colors represent different DEGs.

Expression patterns of DEGs in the key KEGG pathways ECM-receptor interactions, focal adhesion, phagosome, and lysosome KEGG pathways were further evaluated. As shown in Table 5, DEGs related to ECM-receptor interactions and focal adhesion, such as *col5a1*, *itgb7*, and *sptbn5* was up-regulated during the early stage of infection but was down-regulated during the middle and late stages. By contrast, expression of DEGs involved in phagosomes and lysosomes, such as *itgb2*, *srb1*, and *sel1l*, followed the opposite trend, and was down-regulated during the early stage of infection, but up-regulated during the middle and late stages. Comparison of the four KEGG pathways at each time point after infection revealed a significant difference in the expression patterns of these DEGs (Figure 4). 

### 2.7. Key DEGs Identified in the Three Stages of Infection

The more significant DEGs may play important roles in the responses to changes in the environment, hence they were identified and annotated. As shown in Table 6, most of the up-regulated genes between 0 and 8 h post infection such as protein tyrosine phosphatase epsilon (*ptpε*), integrin alpha 7 (*itga7)* and periplakin (*ppl*), are associated with cell signaling, whereas down-regulated genes such as C–X–C motif chemokine 10-like (*cxcl10*) and interferon alpha-inducible protein 27-like (*ifi27l*) are associated with immunity. Genes up-regulated between 8 and 24 hpi are primarily immune-related, including *viperin*, antigen peptide transporter 1 (*tap1*), toll-like receptor 3 (*tlr3*), and interferon regulatory factor-1 (*irf11*). Meanwhile, down-regulated genes such as angiopoietin-related protein 2-like (*angptl2*), phosphoglycolate phosphatase (*pgp*), and RAP1 GTPase activating protein 1 (*rap1gap1*) are mainly related to blood vessels, which may explain why haemorrhage symptoms were observed in grass carp following GCRV infection. Other down-regulated genes include protein damage repair-related genes such as methionine sulfoxide oxidase 3b (*mical3b*) and heat shock protein beta 11 (*hspb11*). Finally, between 24 and 72 h, up-regulated genes are mainly related to programmed cell death, apoptosis, and inflammation, consistent with the high level of cell viability reduction observed at 24 and 72 hpi (Figure 2). The main down-regulated genes are involved in steroid synthesis, while others are associated with microtubules and blood vessels. Details of these DEGs in the three stages are included in Table S4.

### 2.8. Expression Patterns of Key Interferon-Related Genes

**Figure 5:**
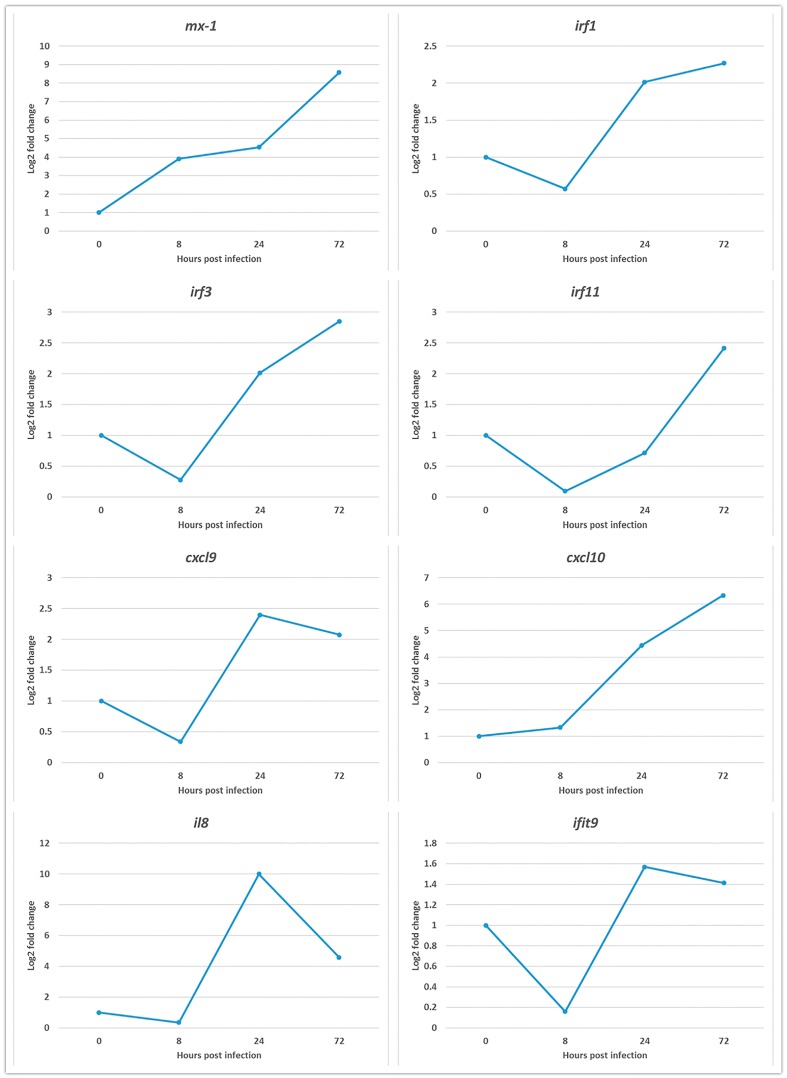
Expression patterns of key genes involved in immune responses. Expression pattern of eight interferon-related genes including interferon-inducible Mx protein 1 (*mx-1*), interferon regulatory factor 1 (*irf1*), interferon regulatory factor 3 (*irf3*), interferon regulatory factor 11 (*irf11*), C–X–C motif chemokine 9 (*cxcl9*), C–X–C motif chemokine 11 (*cxcl10*), interleukin 8 (*il8*), interferon-induced protein with tetratricopeptide repeats 9 (*ifit9*) were examined.

The expression patterns of key interferon-related genes were examined in this study. As shown in Figure 5, except gene *mx-1*, most of the genes showed log2 fold change ≥2 or even ≥10 at 24 or 72 h post-infection, whereas the expression level decreased at 8 h post-infection. These results suggested that strong immune response induced by GCRV infection.

### 2.9. Validation of Selected DEGs by RT-qPCR

**Figure 6:**
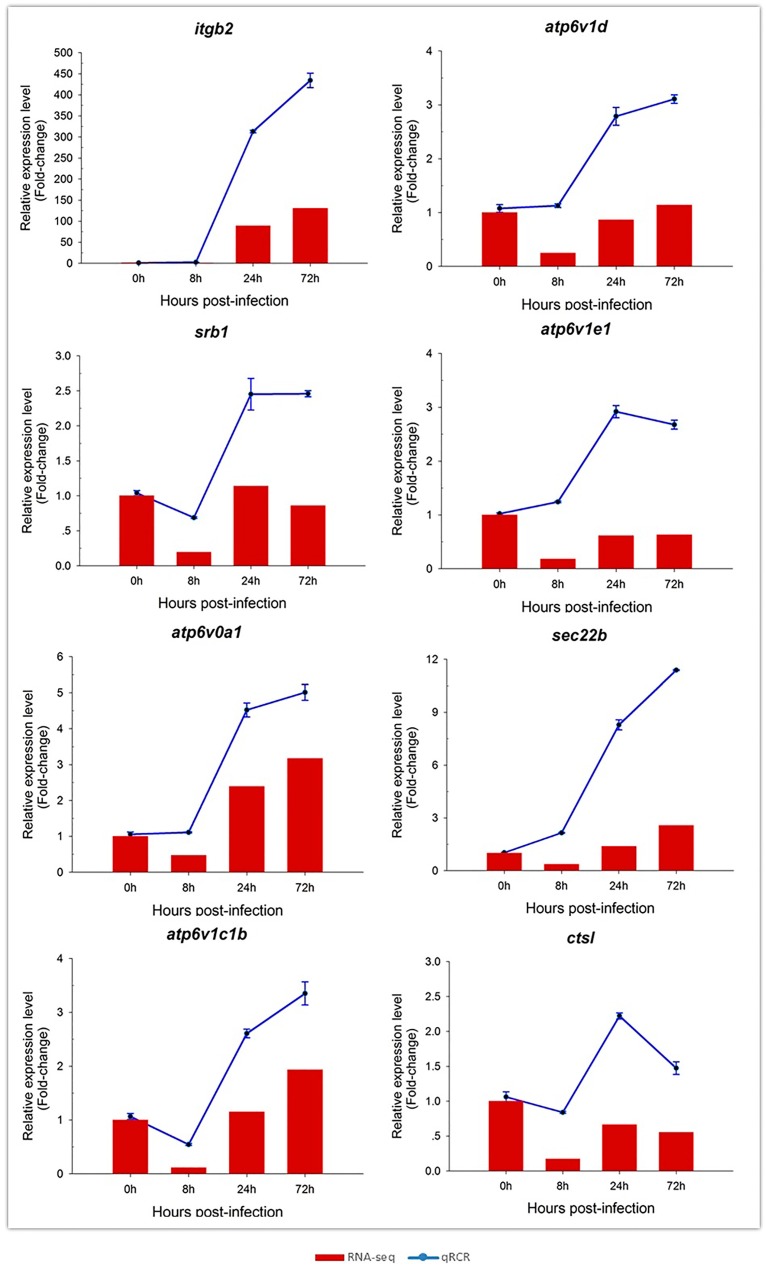
Confirmation of transcriptomics sequencing data by RT-qPCR. Eight DEGs involved in the phagosome pathway were selected for RT-qPCR. These genes included integrin β2 (*itgb2*), V-type proton ATPase subunit D (*atp6v1d*), srb1, V-type proton ATPase subunit E 1-like (*atp6v1e1*), V-type proton ATPase 116 kDa subunit A-like (*atp6v0a1*), sec22b, ATPase, H^+^ transporting, lysosomal, V1 subunit C1b (*atp6v1c1b*), and cathepsin L1-like (*ctsl*). The relative expression levels of the genes at different time points were calculated as the ratio of gene expression level (qPCR) or normalized RPKM level (RNA-seq) relative to that at 0 days (control) post-infection. All data represent the mean ± S.D. of three replicates.

To confirm the reliability of the RNA-Seq data, eight DEGs involved in the phagosome pathway were selected for RT-qPCR analysis. Expression levels of these eight DEGs are shown in Figure 6. Overall, expression patterns of all eight DEGs obtained by qPCR were similar to those from RNA-Seq analysis, although the relative expression levels were not completely consistent. Therefore, the results of the RT-qPCR analysis confirmed the reliability and accuracy of the RNA-Seq data.

## 3. Discussion

At present, previous studies on grass carp transcriptome profiles in response to grass carp reovirus infection have been carried out [10,11,12,13,14]. Most of them involved in the discovery of immune-related genes or uncovered the antiviral immunity mechanism of grass carp. However, the purpose of our study is to understand the whole process of grass carp reovirus infection.

In this study, we used the CIK cells as an in vitro model to explore the molecular changes following reovirus infection. CIK cells were mock-infected or infected with GCRV and harvested at 0 (mock), 8, 24, and 72 hpi. To better understand the process of GCRV entry into cells, it was divided into three stages: early (0–8 h), middle (8–24 h), and late (24–72 h). In each stage, data from the latter time point were compared with that from the former to identify DEGs. The method for DEG identification differed that in the previous study, which used a case-control method [15,16]. DEGs in the 0–8 h were mainly associated with virus adhesion, DEGs in the 8–24 h were largely associated virus endocytosis and transmission, and DEGs in the 24–72 h were involved in cell death and apoptosis. Therefore, the DEGs identified in the present study appear to be representative and accurate.

The CPE appeared as early as 8 h after CIK cells infected with GCRV. DEGs from this stage were mainly enriched with Focal adhesion and ECM-receptor interaction terms. Focal adhesions are sub-cellular structures that mediate the regulatory effects of cells in response to ECM adhesion [17]. In fact, previous studies showed that focal adhesion kinase can interact with rabies virus phosphoprotein P, which is involved in the process of viral infection [18]. During influenza A invasion of cells, inhibition of focal adhesion kinase signaling leads to reduced viral replication [19], suggesting that focal adhesion may be an important pathway during GCRV infection. Moreover, *hspg2*, *ptpε*, and *ppl* were significantly up-regulated during the 0–8 h post infection. HSPG2 plays an important role in mediating virus envelope-target cell interaction during human papillomavirus (HPV) and hepatitis C virus (HCV) infection [20,21]. PTPε plays an important role in the control of macrophage function [22]. And what’s more, tyrosine-protein phosphatase non-receptor type 22 (PTPN22) associates with TNF receptor-associated factor 3 (TRAF3) to augment Toll-like receptor (TLR)-induced type I IFN production [23]. However, PPL acts as a cytolinker between intermediate filament scaffolding and the desmosomal plaque and is involved in epithelial cohesion, intracellular signal transduction and antigen presentation [24]. These results suggest that *hspg2* may be involved in cellular binding of GCRV, after which GCRV likely binds to the receptor on the cell membrane, *ptpε* and *ppl* maybe as signaling molecules that regulate cellular processes.

During the 8 to 24 hpi period, enriched DEGs were mainly related to phagosome and lysosome pathways. In recent years, more and more studies have shown that the viral infection process is closely related to autophagy of host cells. Autophagy can be used as innate immunity and an adaptive immune response against intracellular pathogens [25]. In the phagosome pathway, many genes are up-regulated, including *itgb2*, *srb1*, and *sec22b*, and it is worth noting that the *itgb2* is up-regulated at this period. Cell integrins are commonly used receptors for diverse viral pathogens [26], and reovirus internalization is mediated by integrin β1 proteins, most likely via clathrin-dependent endocytosis [27]. Moreover, integrin β3 proteins serve as receptors of pathogenic hantaviruses [28]. In the phagosome pathway, other receptor genes appear to be up-regulated, such as scavenger receptor *srb1*, which, along with CD81, is the receptor used for entry of the hepatitis C virus into liver cells [29]. The v-SNARE protein SEC22 is involved in the transport of eukaryotic cell endoplasmic reticulum (ER) and Golgi apparatus [30]. Hepatitis C virus may be assembled in the ER and transported to the Golgi apparatus in COPII vesicles to begin Golgi secretion [31]. However, a recent study shows that SEC22 reduces RNAi efficiency by influencing late endosomal functions, through the promotion of fusion between late endosomes and lysosomes [32]. These results suggest that *itgb2* and/or *srb1* may be important for cell entry of GCRV. Furthermore, GCRV entry into the endosomal region followed by fusion with lysosomes is likely to be regulated by *sec22b*.

In the 24–72 h post infection, DEGs were mainly enriched in lipid metabolism, programmed cell death, apoptosis, and inflammation. Genes in the cell death pathway have been studied intensely, and it is interesting to note that genes in the lipid metabolism pathway, such as *cyp51a*, are enriched. CYP51A1 is a cytochrome P450 enzyme, a conserved group of proteins that serve as key players in the metabolism of organic substances and the biosynthesis of important steroids, lipids, and vitamins in eukaryotes [33], and it plays an essential role in mediating membrane permeability [34]. These sterols, located on the plasma membrane of cells, play an important structural role in regulating the fluidity and permeability of membranes while also affecting the activity of enzymes, ion channels and other cellular components [35]. Therefore, these results suggest that GCRV probably not only causes cell death through cell apoptosis and inflammatory pathways but also changes the permeability of the cell membrane by affecting the structure of steroids in the cell membrane, leading to cell death through this mechanism.

## 4. Materials and Methods 

### 4.1. Cells, Virus and Virus Infection

*C. idellus* kidney (CIK) cells purchased from Shenzhen ziker Biological Technology Co., Ltd. (Shenzhen, China) and grown in Medium 199 (Gibco, Gaithersburg, MD, USA) supplemented with 10% fetal bovine serum (FBS), 100 mg/mL penicillin, and 100 mg/mL streptomycin at 28 °C. GCRV was kindly provided by Professor Jianguo Su (Huazhong Agricultural University). CIK cells that grown in 25 cm^2^ flask were mock-infected or infected with GCRV at a multiplicity of infection (MOI) of 1, then harvested at 0 (mock), 8, 24, and 72 h post infection (pi), respectively. Three flasks of cells were collected for each treatment at each time point.

### 4.2. CCK-8 Assay

CCK-8 detection kit (Beyotime, Shanghai, China) was used to measure cell viability according to the manufacturer’s instructions. About 4 × 10^3^ CIK cells were seeded in 96 well plates and cultured in M199 supplemented with 10% FBS at 28 °C for 24 h. Wells containing no cells but only culture medium alone were served as blank control. CIK cells were mock-infected or infected with GCRV at an MOI of 1 and maintained for 0, 8, 24, and 72 h at 28 °C, respectively. Then each well was added 10 μL of CCK-8 solution. After 4 h incubation at 28 °C, the plates were measured spectrophotometrically on Microplate Manager 6 (BIO-RAD, Hercules, CA, USA) at wavelength 450 nm. The relative cell viability was calculated as Ainfected-Ablank-i/A mock-Ablank % (Ablank-i represented the wells containing no cells but only medium and GCRV). Data were presented as means of at least three independent experiments ± standard deviation (S.D).

### 4.3. RNA Isolation, Library Construction, and Sequencing

Cells were collected at 0, 8, 24, and 72 h after infection (C0, C8, C24, and C72 groups, respectively), rapidly frozen in liquid nitrogen, and total RNA was extracted with Trizol reagent (Invitrogen, Waltham, MA, USA). RNase-free DNase treatment was performed in order to remove possible genomic DNA. The DNase was removed by phenol-chloroform re-extraction. mRNA was purified from total RNA using poly-T oligo-attached magnetic beads and segregated into 200–300 bp fragments. The concentration of mRNA used to prepare first strand cDNA is approximate 40 ng/μL and a total of 3 μg mRNA used as input material for first strand DNA synthesis. First strand DNA was synthesized from 6-base random primers and reverse transcriptase using RNA as a template, and second strand cDNA was synthesized using the first strand cDNA as template. In the second strand cDNA synthesis, the base T was replaced by U to generate a chain-specific library. RNA-Seq was performed using chain-specific kits, and chain-specific libraries were used to determine the transcriptional direction of sense and antisense strands to increase the accuracy of subsequent gene function annotation and gene expression analysis.

After library construction, library fragments were enriched by PCR amplification, and selection based on fragment size resulted in a 300–400 bp library. The library was tested using an Agilent 2100 Bioanalyzer (Agilent, Palo Alto, CA, USA), and both the total and effective concentration of the library were tested. Based on the effective concentration and the amount of data required, libraries containing different Index sequences (samples plus different Index sequences, and difference data of samples according to Index) were mixed proportionally. The mixed library was diluted to 2 nM and alkaline degeneration was performed to generate a single-chain library. Next, 150 bp pair-end reads were performed on the library using an Illumina NextSeq500 sequencing platform to carry out Next-Generation Sequencing (NGS) at Personalbio (Personalbio, Shanghai, China). For each treatment in each time point, the sample was sequenced three times as three technical replicates.

### 4.4. Data Analysis

Clean data were obtained by removing adapters, poly-N sequences and poor-quality data [36]. The Q20, Q30, and GC content of clean data were calculated, and all downstream analysis was performed using high-quality clean data.

Clean data were mapped to the grass carp reference genome [37] using TopHat2 software [38]. The number of reads mapped to each gene was used HTSeq software [39], and the reads per kilobase of the exon model per million mapped reads (RPKM) was calculated as expression values for each gene [40].

### 4.5. Differential Expression Analysis

Differential expression analysis using data collected at different time points was performed using the DESeq package [41]. The resulting *p*-values were adjusted using the Benjamini and Hochberg approach to control the false discovery rate. Genes with an adjusted *p*-value < 0.05 (*q*-value < 0.05) and fold change > 2 in DESeq analysis were assigned as differentially expressed genes (DEGs). 

ClueGO and CluePedia were used to perform Gene Ontology (GO) enrichment analysis [42,43]. In GO enrichment analysis, only categories with a *p*-value < 0.05 were considered as enriched in the network.

The Kyoto Encyclopedia of Genes and Genomes (KEGG) database was used to indicate the location of the DEGs in the different pathways. In this study, KOBAS software was used to test the statistical enrichment of DEGs in KEGG pathways [44]. Pathways with *p*-values < 0.05, were considered for as statistical significance.

### 4.6. Validation of DEGs by RT-qPCR

To confirm the reliability of the data obtained with RNA-Seq, eight DEGs were selected for RT-qPCR using the primers listed in Table S5. Primers were designed using the Primer Premier 5.0 software (PREMIER Biosoft International, Palo Alto, CA, USA) and only with an efficiency of 90–110% were used. First strand cDNAs were obtained from total RNA (>3 μg) using a random hexamer primer and the ReverTra Ace kit (TOYOBO, Osaka, Japan). RT-qPCR was performed using a fluorescence quantitative PCR instrument (BIO-RAD). Each RT-qPCR mixture contained 0.8 μL of each primer, 1 μL template, 10 μL 2× SYBR Green master mix (TOYOBO), and 7.4 μL ddH_2_O. The program for RT-qPCR was 95 °C for 10 s, followed by 40 cycles at 95 °C for 15 s, 60 °C for 15 s, and 72 °C for 30 s. Three replicates were included for each sample, and the *β-actin* gene of grass carp was served as an internal control to normalize the expression levels. Relative expression levels of the different genes were calculated using the 2^−ΔΔ*C*t^ method [45]. All data represent the mean ± standard deviation of three replicates.

## 5. Conclusions

In conclusion, the results of this study provide insight into the mechanisms of grass carp reovirus infection. In the early stage of infection, GCRV interacts with polysaccharides and accumulates on the cell surface, then binds to the GCRV receptor to initiate endocytosis mediated by integrins. In the middle stage of infection, GCRV is likely to be regulated by v-SNARE protein and transported from the endosomal region to lysosomes. In the last stage, GCRV functions through several different pathways, by affecting the structure of steroids in the cell membrane, then leading to cell death in the final stage. These findings broaden our understanding of the invasion of GCRV and may assist the development of strategies to combat diseases caused by GCRV.

## Figures and Tables

**Table 1:** Summary of sequencing data obtained in this study.

Sample	Raw Reads	Clean Reads	Clean Data (bp)	Q20 (%)	Q30 (%)	Mapped (%)
C0	47,178,538	46,732,668	6,905,828,036	97.24	93.27	87.96
C8	45,666,538	45,217,760	6,679,552,386	96.98	92.7	87.24
C24	41,778,528	41,398,026	6,111,817,194	97.21	93.15	88.07
C72	41,012,162	40,628,112	6,014,381,958	97.15	93.07	87.40

**Table 2:** Summary of identified DEGs.

Case	Control	Up-Regulated Genes	Down-Regulated Genes	Total DEGs
C8	C0	35	41	76
C24	C8	463	90	553
C72	C24	150	134	284

**Table 3:** Results of GO enrichment during the three stages following infection.

Time	Category	GO_Term	Gene Num.
0−8 h	Biological_process	Iron ion homeostasis	3
	Cellular_component	Inclusion body	2
	molecular_function	FAD binding	2
	biological_process	Transition metal ion homeostasis	3
	Biological_process	Carbohydrate biosynthetic process	3
8−24 h	Cellular_component	Vacuole	16
	Molecular_function	Iron ion binding	13
	Biological_process	Organic acid biosynthetic process	12
	Molecular_function	Oxidoreductase activity	13
	Cellular_component	Lysosome	10
24−72 h	Biological_process	Lipid biosynthetic process	19
	Biological_process	Steroid metabolic process	11
	Biological_process	Alcohol biosynthetic metabolic process	8
	Biological_process	Isoprenoid biosynthetic process	6
	Biological_process	Steroid metabolic process	8

**Table 4:** KEGG enrichment results of the three stages following infection.

Time	Pathway	DEGs	*p*-Value
0−8 h	ECM-receptor interaction	3	0.002216
	Focal adhesion	4	0.005585
	Mineral absorption	2	0.008391
	Glutathione metabolism	2	0.012446
	Cytokine-cytokine receptor interaction	3	0.01791
8−24 h	Collecting duct acid secretion	4	0.0017689
	Lysosome	10	0.0019208
	Phagosome	10	0.0023994
	Hippo signaling pathway	11	0.0052756
	Sphingolipid metabolism	5	0.0138641
24−72 h	Steroid biosynthesis	11	1.58 × 10^−16^
	Terpenoid backbone biosynthesis	7	7.96 × 10^−9^
	Metabolism of xenobiotics by cytochrome P450	5	0.000122
	Sesquiterpenoid and triterpenoid biosynthesis	2	0.000187
	Carbon fixation pathways in prokaryotes	3	0.001499

**Table 5:** DEGs associated with four major pathways.

Pathway	Gene Name	Log2 Fold Change		
		8 h	24 h	72 h
ECM-receptor interaction	*col5a1* (collagen_alpha-1(V)_chain)	1.27	0.79	−0.49
	*itgb7* (integrin_beta-7-like)	1.52	−0.40	−0.42
	*itga7* (integrin_alpha-7)	0.76	−0.16	0.27
Focal adhesion	*col5a1* (collagen_alpha-1(V)_chain)	1.27	0.79	−0.49
	*itgb7* (integrin_beta-7-like)	1.52	−0.40	−0.42
	*itga7* (integrin_alpha-7)	0.76	−0.16	0.27
	*sptbn5* (spectrin_beta_chain,_non-erythrocytic_5-like)	0.77	−0.09	−0.31
Lysosome	*arsd* (arylsulfatase_D_isoform_X2)	−0.69	1.13	1.30
	*glb1l2* (beta-galactosidase-1-like_protein_2-like)	−1.83	1.21	1.49
	*Asah1* (*N*-acylsphingosine_amidohydrolase_1_precursor)	−1.15	1.06	1.54
	*neu3.2* (Sialidase-3-like)	−1.01	1.29	1.15
	*arsk* (arylsulfatase_K_isoform_X1)	−0.75	0.83	0.19
	*atp6v0a1* (V-type proton ATPase 116 kDa subunit a-like)	−1.06	1.23	1.54
	*sel1l* (Protein sel-1 homolog 1 precursor)	−0.43	1.05	1.20
	*abcg2c* (ATP binding cassette subfamily G member 2)	−1.25	1.58	1.83
	*ctsc* (dipeptidyl_peptidase_1_precursor)	−1.13	0.83	0.47
	*dnase2* (deoxyribonuclease-2-beta-like)	−1.06	0.97	1.13
Phagosome	*itgb2* (Integrin beta-2-like)	−1.73	2.33	2.76
	*atp6v1d* (V-type proton ATPase subunit D)	−1.00	0.79	1.09
	*srb1* (scavenger receptor class B member 1)	−0.81	0.83	0.44
	*atp6v1e1* (V-type proton ATPase subunit E 1-like)	−1.09	0.68	0.63
	*atp6v0a1* (V-type proton ATPase 116 kDa subunit a-like)	−1.06	1.23	1.54
	*sec22b* (Vesicle-trafficking protein SEC22b-A-like)	−0.93	0.85	1.62
	*atp6v1c1b* (ATPase, H^+^ transporting, lysosomal, V1 subunit C1b)	−1.90	1.18	1.82
	*ctsl* (Cathepsin L1-like)	−1.13	0.83	0.47

**Table 6:** Top five up-regulated and down-regulated genes of the three stages following infection.

Time Period		Gene Name	Log2 Fold Change	Description
0−8 h	Up	*ptp* *ε*	6.01	Protein tyrosine phosphatase epsilon
		*itga7*	5.49	Integrin alpha7
		*hspg2*	4.64	Heparan sulfate proteoglycan 2
		*leprel4*	4.12	Synaptonemal complex protein SC65
		*ppl*	3.86	Periplakin
	Down	*cxcl10*	−7.11	C–X–C motif chemokine 10-like
		*tnfrsf9*	−5.51	Tumour necrosis factor receptor superfamily member 9-like
		*plaur*	−5.39	Urokinase plasminogen activator surface receptor-like
		*ubl1*	−4.71	Ubquitin-like protein 1
		*ifi27*	−4.63	Interferon alpha-inducible protein 27-like
8−24 h	Up	*itgb2*	6.84	Integrin beta-2
		*viperin*	4.71	Virus inhibitory protein
		*tap1*	3.81	Antigen peptide transporter 1
		*tlr3*	3.05	Toll-like receptor 3
		*irf11*	2.97	Interferon regulatory factor 11
	Down	*angptl2*	−5.78	Angiopoietin-related protein 2-like
		*pgp*	−5.22	Phosphoglycolate phosphatase
		*mical3b*	−4.9	Protein-methionine sulfoxide oxidase mical3b
		*hspb11*	−4.45	Heat shock protein beta-11
		*rap1gap1*	−2.73	rap1 GTPase-activating protein 1
24−72 h	Up	*tnfrsf9*	2.68	Tumour necrosis factor receptor superfamily member 9
		*hspa*	2.42	Heat shock 70 kDa protein
		*fbw7*	2.35	F-box/WD repeat-containing protein 7
		*nfil3*	1.87	Nuclear factor, interleukin 3 regulated
		*osbp1*	1.57	Oxysterol-binding protein 1
	Down	*lss*	−2.95	Lanosterol synthase
		*sc5d*	−2.19	Lathosterol oxidase
		*cyp51a1*	−1.9	Lanosterol 14α-demethylase
		*ablim1*	−1.11	Actin-binding LIM protein 1
		*cyr61*	−1.08	Cyr61 protein

## Supplementary Materials

Supplementary materials can be found at http://www.mdpi.com/1422-0067/19/2/488/s1.
